# Antibiotics-Induced Intracranial Hypertension: A Case Report With Literature Review

**DOI:** 10.7759/cureus.55424

**Published:** 2024-03-03

**Authors:** Mariam Assardoun, Yahya Naji, Soumia Nedday, Sara Laadami, Nawal Adali

**Affiliations:** 1 Department of Neurology, Agadir University Hospital, Agadir, MAR; 2 Department of Neurology, Neurosciences Innovation Cognition Ethique (NICE) Research Team, Rein Endocrinologie Gastroentérologie Neurosciences Ethique (REGNE) Research Laboratory, Faculty of Medicine and Pharmacy, Ibn Zohr University, Agadir, MAR

**Keywords:** csf pressure, cerebral mri, antibiotics, headache, intracranial hypertension

## Abstract

Idiopathic intracranial hypertension (IIH) is a rare condition characterized by increased intracranial pressure, with an unknown cause. However, the pathophysiology of antibiotic-induced IIH remains unclear. The clinical symptoms include headache, visual disturbances, and vomiting. The diagnosis is confirmed by an elevated intracranial pressure (ICP) with normal CSF study and cerebral imaging. Management includes discontinuing the offending antibiotic and reducing ICP with medications such as acetazolamide or diuretics. Therefore, surgical intervention may be necessary in severe cases.

In this article, we report the case of a 19-year-old patient, admitted with symptoms of intracranial hypertension syndrome, occurring three days after receiving antibiotics (gentamicin, penicillin). Physical examination revealed bilateral optic disc edema. Cerebral magnetic resonance imaging (MRI) revealed indirect signs of intracranial hypertension. The CSF pressure measurement was approximately 290 mmHg, while CSF and other laboratory blood tests were normal. The patient received methylprednisolone bolus and topiramate (50 mg/day). A month later, the clinical outcome showed regression of headaches and regression of the papilledema.

## Introduction

Idiopathic intracranial hypertension (IIH) is a rare disorder that occurs in 1-2 cases per 100,000 people per year [1,2]. It is characterized by headache, papilledema, and elevated intracranial pressure, with normal brain MRI and cerebrospinal fluid (CSF) findings. Several drugs have been implicated as causes of IIH, including antibiotics [3,4]. Antibiotics are a group of drugs used for the treatment of bacterial infections and are divided into several classes, including beta-lactams, aminoglycosides, and tetracyclines.

Several medications have been reported to cause intracranial hypertension, but few antibiotics have been implicated in this pathology [3]. According to the literature, intracranial hypertension is a well-recognized side effect of tetracyclines [3]. To our knowledge, this case is the first to report IIH following a combination of penicillin and gentamicin.

## Case presentation

**Figure 1:**
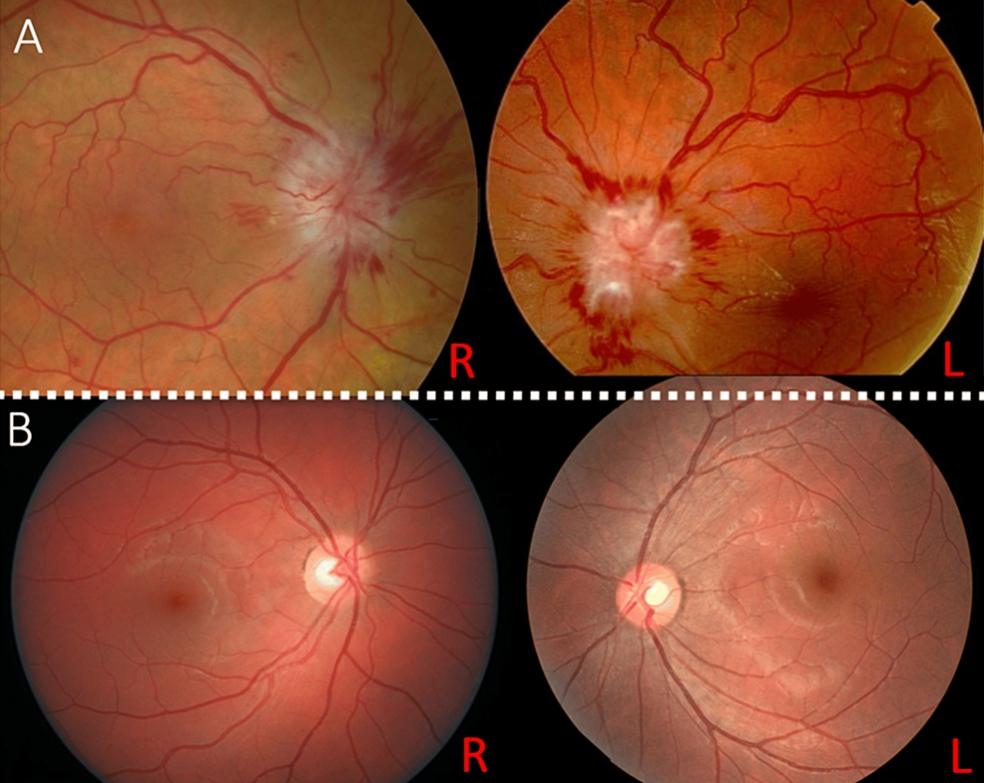
Colour fundus photographs A: Bilateral papilledema at the time of diagnosis B: Resolution of papilledema four weeks later

We report the case of a 19-year-old female patient with no history of chronic kidney disease, hypothyroidism, hyperthyroidism, anemia, or contraceptive treatment. She was admitted to the trauma unit for a gluteal abscess five days after penicillin injection, where she benefited from surgical drainage with antibiotic coverage (gentamicin and penicillin). Three days later, the patient suddenly presented with complete intracranial hypertension syndrome consisting of binocular horizontal diplopia, decreased visual acuity, headache, and vomiting. A neurological assessment was requested, which found a conscious patient with a normal tone, muscle bulk, and power in her upper and lower extremities. Examination by an ophthalmologist revealed normal visual acuity (20/20 in both eyes). Her anterior segment examination result was normal, although the fundus examination showed moderate bilateral optic disc edema (Figure 1).

**Figure 2:**
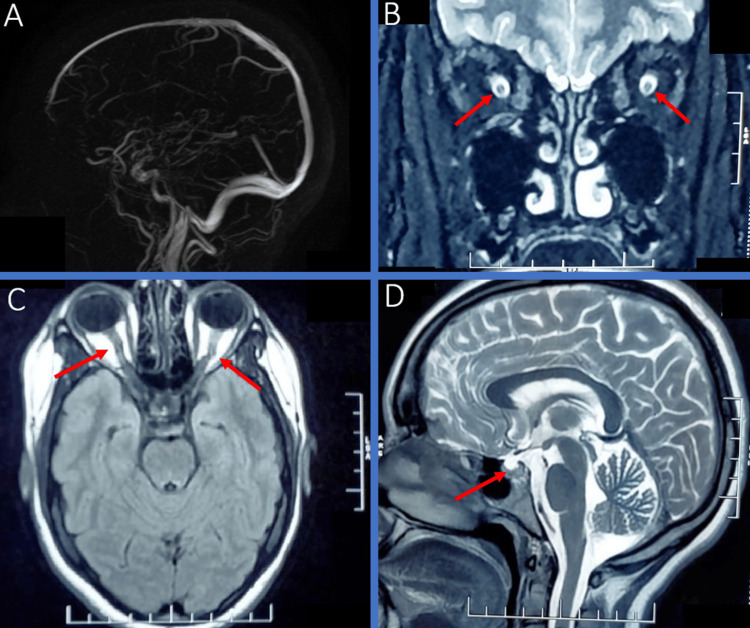
Brain MRI of our patient showing indirect signs of IIH A: Normal MR venography B: Coronal T2, widening of the optic nerve sheaths C: FLAIR, tortuous optic nerves D: Sagittal T2, empty sella IIH: idiopathic intracranial hypertension FLAIR: fluid-attenuated inversion recovery

The cerebral magnetic resonance imaging (MRI) revealed indirect signs of intracranial hypertension (tortuous optic nerves, widening of the optic nerve sheaths, empty sella, and collapsed ventricles without cerebral thrombophlebitis) (Figure 2).

Laboratory evaluation of the complete blood cell count, biochemical tests (blood urea nitrogen, creatine, sodium, potassium, phosphocalcic, and glucose), serum protein electrophoresis, blood hepatitis B and C, HIV serology, antinuclear antibody titer, and thyroid tests were normal. CSF pressure measurement was approximately 290 mmHg, while the CSF study results were normal (cell count: four cells; protein at 0.40 g/l). Bacterial and viral markers in the CSF were negative. Penicillin and gentamicin were stopped, and the patient received a methylprednisolone bolus (1 g/day for three days) and acetazolamide (750 mg/day). Two days after acetazolamide administration, the patient developed distal paresthesia and vomiting, which led to a switch to topiramate (50 mg/day). One month later, the clinical outcome showed a regression of headaches and the disappearance of papilledema (Figure 1).

The absence of direct causative evidence, temporal relationship, and support cases in the literature collectively provide a strong basis for our assertion of antibiotic-associated intracranial hypertension with penicillin and gentamicin.

## Discussion

Idiopathic intracranial hypertension can usually be diagnosed based on the modified Dandy criteria. IIH can be expressed by classic symptoms such as headache, vomiting, and papilledema. Cerebral MRI shows indirect signs of IIH, such as collapsed ventricles, an empty sella, or a prominent cisterna magna. Lumbar puncture confirmed elevated opening pressure in a normal CSF study [5,6].

IIH’s pathogenesis is not fully understood, but it is strongly associated with risk factors, such as obesity and medication [7,8]. Many medications can cause IIH; the most implicated are estrogens, tetracycline, corticosteroids, and vitamin A. Most reported cases did not impute the IIH to medication on the basis of chronology appearance, duration of drug intake, and the presence of similar reports in the literature [9]. Our literature review found that gentamicin was retained as a possible cause of IIH, and another case incriminated penicillin a week after its administration. Both cases reported a regression of headaches and a decrease in intracranial pressure a few days after discontinuation of medication [10,11]. To the best of our knowledge, our case is the first to be characterized by the association of gentamicin and penicillin as a possible cause of IIH.

The management of drug-induced IIH includes stopping the offending treatment and using acetazolamide or topiramate to decrease the CSF pressure. However, in refractory cases with a risk of visual loss, surgical therapy with optic nerve sheath fenestration or lumbo-peritoneal shunt may be indicated [6,12]. Our patient was treated with topiramate with good recovery and no relapse after six months of follow-up.

## Conclusions

Our case sheds light on the critical association between certain antibiotics and the development of idiopathic intracranial hypertension. The presented case underscores the importance of vigilance among healthcare professionals in recognizing and managing this rare yet potentially serious side effect. Clinicians should consider antibiotic-induced intracranial hypertension as a differential diagnosis in patients presenting with neurological symptoms during antibiotic therapy. Timely recognition and intervention are crucial to prevent further complications and ensure optimal patient outcomes. Further research is warranted to elucidate the underlying mechanisms and risk factors, contributing to a better understanding of this uncommon but significant clinical entity.
